# Recent clinical practice guidelines for the management of low back pain: a global comparison

**DOI:** 10.1186/s12891-024-07468-0

**Published:** 2024-05-01

**Authors:** Tianyu Zhou, David Salman, Alison. H. McGregor

**Affiliations:** 1 MSk lab, Department of Surgery & Cancer, Imperial College London, London, 2nd Floor, Sir Michael Uren Hub, 86 Wood Lane, W12 0BZ UK; 2 Department of Primary Care and Public Health, Imperial College London, London, W6 8RP UK

**Keywords:** Clinical practice guidelines, Low back pain, Management, Treatment, Review

## Abstract

**Background:**

Low back pain (LBP) is a significant health problem worldwide, with a lifetime prevalence of 84% in the general adult population. To rationalise the management of LBP, clinical practice guidelines (CPGs) have been issued in various countries around the world. This study aims to identify and compare the recommendations of recent CPGs for the management of LBP across the world.

**Methods:**

MEDLINE, EMBASE, CINAHL, PEDro, and major guideline databases were searched from 2017 to 2022 to identify CPGs. CPGs focusing on information regarding the management and/or treatment of non-specific LBP were considered eligible. The quality of included guidelines was evaluated using the Appraisal of Guidelines for Research and Evaluation (AGREE) II instrument.

**Results:**

Our analysis identified a total of 22 CPGs that met the inclusion criteria, and were of middle and high methodological quality as assessed by the AGREE II tool. The guidelines exhibited heterogeneity in their recommendations, particularly in the approach to different stages of LBP. For acute LBP, the guidelines recommended the use of non-steroidal anti-inflammatory drugs (NSAIDs), therapeutic exercise, staying active, and spinal manipulation. For subacute LBP, the guidelines recommended the use of NSAIDs, therapeutic exercise, staying active, and spinal manipulation. For chronic LBP, the guidelines recommended therapeutic exercise, the use of NSAIDs, spinal manipulation, and acupuncture.

**Conclusions:**

Current CPGs provide recommendations for almost all major aspects of the management of LBP, but there is marked heterogeneity between them. Some recommendations lack clarity and overlap with other treatments within the guidelines.

**Supplementary Information:**

The online version contains supplementary material available at 10.1186/s12891-024-07468-0.

## Introduction

Low back pain (LBP) is one of the most common musculoskeletal conditions globally. According to the Global Burden of Disease Study, LBP continues to be the leading cause of years lived with disability [1], increasing from 42.5 million to 64.9 million globally between 1990 and 2017 [2]. The point prevalence of LBP has been estimated to be as high as 18%, resulting in increased activity limitation and absenteeism from work [2]. LBP not only affects individuals’ daily lives but also imposes a heavy social burden and economic cost, representing a huge challenge to healthcare systems. This is now as apparent in low-income countries as it is in the more affluent and developed countries across the world [3]. Given the prevalence of LBP, healthcare professionals managing LBP need access to up-to-date, evidence-based information to assist them in treatment decision-making [4]. To standardise the management of LBP, professional bodies have developed an increasing number of clinical practice guidelines (CPGs), providing recommendations for diagnosis and management [4].

There has been considerable growth in LBP management CPGs since the first LBP guideline was published in 1987 by the Quebec Task Force [5]. Over the last few decades, various institutions within different countries have issued LBP guidelines, and an ever-expanding repository of publications on CPGs has emerged, with potentially conflicting recommendations [6, 7]. CPGs have also shifted from being built primarily based on expert opinion in the past, to being more evidence-based, including increasingly sophisticated methodologies and implementation strategies [8, 9]. However, numerous, sometimes differing, and occasionally contradictory guidelines will further complicate the selection of treatments for healthcare professionals. Indeed, poor guidelines lead to ineffective interventions, inefficient use of scarce resources, and potentially patient harm [10].

Although previous studies have reviewed clinical recommendations for managing LBP in general [11, 12], researchers indicated the need to place additional emphasis on differentiating between acute, subacute, and chronic LBP management. In light of the evolving nature of evidence, it is important to investigate the level of consensus among recently updated or developed CPGs concerning treatment recommendations for LBP across different durations. This study aims to identify and summarise the recommendations of recent CPGs for treating and managing LBP of different duration across the world.

## Methods

### Search strategy

Since guidelines are updated every three to five years, an original search of guidelines was performed in March 2022 for the period 2017–2021, then repeated in January 2023 to ensure any relevant latest published or updated CPGs in 2022 were included. CPGs were searched using a systematic approach, including a structured and unstructured search. The structured search was initially conducted using MEDLINE, EMBASE, CINAHL, and PEDro with the following keywords: low(er) back pain, chronic pain, clinical practice guideline*, practice guideline*, and clinical guideline*. As CPGs are rarely published in medical journals and databases [13], an unstructured search was conducted to identify additional guidelines from the following guideline organisation databases: the National Guideline Clearinghouse, the Guidelines International Network, the Trip medical database, the Agency for Clinical Innovation, the World Health Organization, the Latin American and Caribbean Health Sciences Literature, the National Institute for Clinical Excellence, and the Scottish Intercollegiate Guidelines Network. We also identified relevant CPGs for LBP management by utilising citation monitoring and reviewing reference lists from key guideline reviews. Furthermore, consultations with professionals were undertaken to ascertain any CPGs that may have been inadvertently omitted in our initial search. Supplementary Material 1 illustrates the search strategy.

### Eligibility criteria

Criteria for inclusion in the review were: (1) the CPG was issued by a national body or international federation, (2) the CPG stated specific recommendations on the clinical management of non-specific LBP, (3) the CPG concerned adult populations (18 years or over), and (4) the CPG was published or updated from 2017 to 2022. Exclusion criteria were: (1) the consensus or summary only, (2) the CPG only focused on non-therapeutic interventions (e.g., prevention, diagnosis), (3) the CPG only addressed specific approaches (i.e., pharmacological and chiropractic guidelines), (4) a previous version of an updated guideline, and (5) LBP CPG’s targeting a specific pathology e.g. radiculopathy. The CPGs were not limited by country of origin, and no language restrictions were applied. If multiple guidelines were identified by different governing bodies from the same country, all identified guidelines were included. We used the most up-to-date version when more than one guideline was published by the same governing body. When guidelines existed in both English and the language of the country of origin from the same governing body, the English publication of the guideline was used.

### Study selection

The titles and abstracts were initially screened for eligibility by one of the reviewers (TZ). After the preliminary screening phase, the selected publications’ full texts were retrieved and reviewed. The other two reviewers (AM and DS) checked this process at each stage and were consulted when discrepancies persisted.

### Quality assessment

All included guidelines were appraised for methodological quality using the Appraisal of Guidelines for Research and Evaluation (AGREE) II instrument [14]. The AGREE II tool is a reliable and valid generic tool used to assess the methodological quality of clinical guidelines [15]. The AGREE II tool consists of 23 items organised in six domains: scope and purpose, stakeholder involvement, rigour of development, clarity of presentation, applicability, and editorial independence, plus two overall assessments. Each AGREE II domain is rated on a 7-point scale ranging from strong disagreement (1 point) to strong agreement (7 points). Domain scores are calculated by summing up all the scores of the individual items in a domain and by scaling the total as a percentage of the maximum possible score for that domain.

To date, a validated threshold for distinguishing high, medium, or low-quality guidelines is still lacking for the AGREE II checklist [16]. After scoring all the guidelines reviewed for this study, a consensus was reached among the authors: guidelines that scored more than 75% on average [17] or global rating ≥ 6 points [18] were deemed “high” quality. After completing training on the use of the AGREE II guidelines, an independent reviewer (TZ) appraised all guidelines. Assessments were then reviewed by authors (AM & DS).

### Data extraction

The following data were extracted from each guideline using a standardised form: country of publication, year of publication, organisation that published the CPG, duration of LBP, classification of LBP in the guideline, and recommendations regarding treatment. The methodologies employed for grading the quality of evidence, and the evidence underpinning the recommendations were also considered. In addition to the core data extraction parameters, we documented the intended target audience for each CPG, the evolutionary approach to the CPG, the composition of the multidisciplinary expert panel in the guideline development, and the extent of patient involvement. The website of each developer was also accessed in case any relevant documents were missing. One reviewer (TZ) independently performed data extraction from each guideline, and the other two reviewers (AM and DS) were responsible for checking this process with any disagreements resolved through discussion.

### Data synthesis

Recommendations from all guidelines were synthesised according to whether an intervention is (1) recommended; (2) not recommended; (3) no evidence; or (4) not mentioned. If the guideline used the following terminology: ‘consider’, ‘offer’, ‘provide’, ‘endorse’, ‘should advice’, ‘should receive’, ‘should suggest’, ‘effective evidence’ ‘should/may/can be used’, ‘is effective’ (or similar wording), the intervention was rated as “recommended”. If the guideline used the following terminology: ‘not suggest/advice’, ‘not support’, ‘not recommend’, ‘not effective’, ‘not improve’, ‘no recommendation’, ‘no benefit’, ‘suggest against’, ‘endorse against’, ‘have an unfavourable benefit/risk’ (or similar wording), the intervention was rated as “not recommended”. If the guideline used the following terminology: ‘insufficient’, ‘‘inconclusive’, ‘no convincing evidence’, ‘conflicting evidence’, ‘is unclear’ (or similar wording), the intervention was rated as ‘no evidence’ to make a recommendation’. If the intervention in the guidelines was not referred to, this intervention was rated as “not mentioned”. We stratified recommendations by the duration of LBP (i.e., acute LBP, subacute LBP, chronic LBP, and unspecified duration of LBP) based on what the guidelines were specifying themselves.

## Results

### Selection of guidelines

**Fig. 1:**
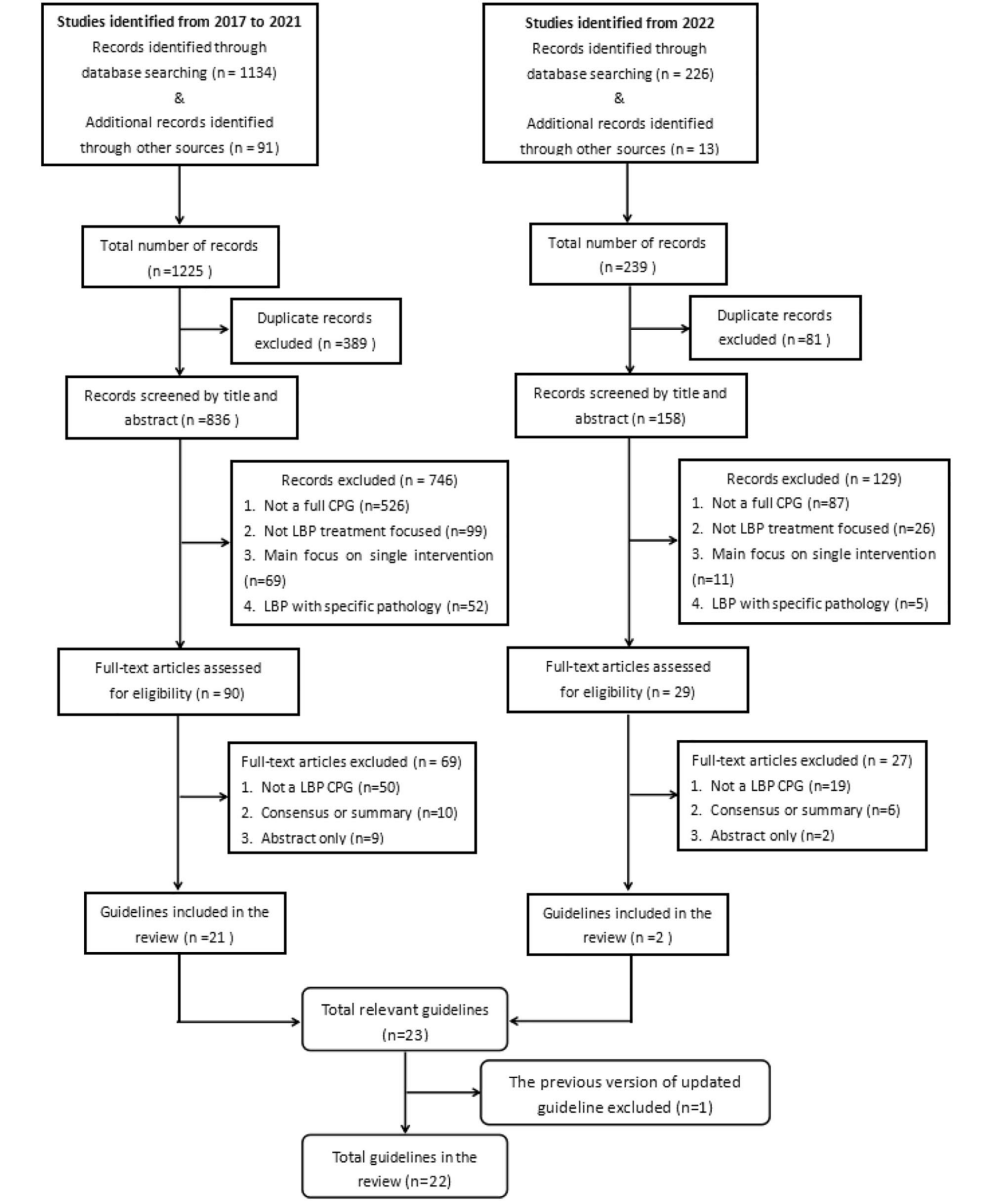
Flow chart of guideline selection process

The search retrieved 1134 citations from medical databases and an additional 91 citations from guideline organisation databases. After removing duplicates, a total of 836 citations underwent titles/abstracts screening. Of those, 746 citations were eliminated, resulting in 90 full-text items warranting further consideration. After full-text screening, 21 CPGs were included in the review. The subsequent search found a total of 239 studies, of which two CPGs were updated in 2022. Finally, 22 CPGs were deemed eligible and included in this review. Fig. 1 shows the number of studies at each stage of selection and the excluded records during the selection process.

### Guideline characteristics

The 22 contemporary CPGs originate from the following 15 countries and regions: Denmark [19], United States [20–25], Peru [26], Austria [27], Canada [28, 29], Germany [30], Philippines [31], Qatar [32], Belgium [33], the UK [34], France [35], Netherlands [36, 37], Russia [38], Japan [39], and Scotland [40]. Five guidelines were issued by different professional associations from the United States, including the American College of Physicians (ACP) [20], Veterans Affairs/Department of Defense (VA/DoD) [21], Institute for Clinical Systems Improvement (ICSI) [22], American College of Occupational and Environment (ACOEM) [23], North American Spine Society (NASS) [24], and Academy of Orthopaedic Physical Therapy (AOPT) [25], two guidelines from Canada, including Toward Optimized Practice (TOP) [28] and Patients Experience Evidence Research (PEER) [29], and two guidelines from Netherlands, including Dutch General Practitioners Association (NHG) [36] and Royal Dutch Society for Physiotherapy (KNGF) [37].

Nine guidelines (41%) targeted recommendations regarding acute, subacute, and chronic LBP (USA-VA/DoD [21], USA-NASS [24], USA-ACOEM [23], USA-ACP [20], Austria [27], Canada-TOP [28], Germany [30], Philippines [31], and Qatar [32], two guidelines (9%) addressed both acute and subacute LBP (USA-ICSI [22] and Peru [26]), and one guideline (5%) considered both acute and chronic LBP (USA-AOPT [25]). One guideline (5%) focused on acute LBP (Denmark [19]), and four guidelines (18%) focused on chronic LBP (Russia [38], Japan [39], Canada-PEER [29] and Scotland [40]), respectively. In addition, five guidelines (23%) provided recommendations regardless of the duration of symptoms (Belgium [33], the UK [34], France [35], the Netherlands-NHG [36], and the Netherlands-KNGF [37]).

Each guideline considered the duration of symptoms, but they varied in scope and by definition. Six guidelines defined acute LBP as less than four weeks duration (USA-ICSI [22], USA-ACOEM [23], USA-ACP [20], USA-VA/DoD [21], Austria [27], and Philippines [31]), whilst eight guidelines specified less than six weeks duration (Peru [26], USA-AOPT [25], USA-NASS [24], Germany [30], Qatar [32], Belgium [33], the Netherlands-NHG [36], and the Netherlands-KNGF [37]). Eight of the guidelines characterised acute and subacute LBP as having a duration of less than 12 weeks but without specific cutoff points to distinguish between the two. (Denmark [19], Canada-TOP [28], the UK [34], France [35], Russia [38], Canada-PEER [29], Japan [39], and Scotland [40]). All guidelines defined chronic LBP as more than 12 weeks in duration.

Sixteen of the CPGs explicitly detailed the involvement of experts across various disciplines. Specifically, the involvement of various healthcare professionals was as follows: physiotherapists were included in 17 CPGs [19, 21, 22, 24–26, 28–35, 37, 39, 40], general practitioners in 15 [19, 21, 22, 24, 26–30, 32–35, 37, 40], manual therapists [19, 21, 22, 24, 27, 28, 32, 37, 39] and psychologists [21, 24, 27–30, 34, 35, 40] in 9 each, pain management specialists [21, 27, 29, 32, 34, 35, 39] and radiologists [19, 21, 22, 24, 27, 28, 35] in 7 each, rheumatologists [19, 26, 27, 30, 32] and nurses [21, 29, 32, 34, 40] in 5 each, and neurosurgeons [24, 27, 30] as well as surgical spine specialists [29, 32, 33] were included in 3 CPGs each.

All 22 CPGs showed explicit information about the evidence. Most guidelines were based on varied evidence, including previously issued guidelines, systematic reviews (SRs), randomised controlled trials (RCTs), observational studies, or expert opinion. More specifically, eleven CPGs considered the previous CPGs in their review of the evidence [19, 22, 24–26, 28, 31, 33, 35, 37, 39], twenty-one included prior SRs with or without meta-analysis [19, 21–40], all CPGs included RCTs [19–40], thirteen included observational studies [21, 22, 24, 27, 28, 30–32, 34–36, 39, 40], and nine CPGs included expert opinion or formal consensus [27, 28, 30–33, 35, 37, 40]. Supplementary Material 2 describes details of the characteristics of the selected guidelines.

### Quality assessment of included guideline

Table 1 presents average scaled scores and overall assessments for each CPG from AGREE II. The overall quality of CPGs was moderately variable, with mean scaled scores ranging from 42 to 86%. The average overall assessment score of the selected guidelines was 5.0, ranging from 3 to 7. Seven guidelines scored 75% on average or overall personal rating ≥ 6 points were deemed high quality, including Denmark [19], US-AOPT [25], USA-ICSI [22], USA-VA/DoD [21], USA-ACP [20], Belgium [33], and the UK [34]. No guideline was deemed to be of low quality.

**Table 1:** The methodological quality of clinical practice guidelines with AGREE II

Country	Domain 1: Scope and Purpose	Domain 2: Stakeholder Involvement	Domain 3: Rigour of Development	Domain 4: Clarity of Presentation	Domain 5: Applicability	Domain 6: Editorial Independence	Mean scaled score	Overall assessment	
								First global rating (Personal rating)	Second global rating (I would recommend?)
**Denmark (2019)** *	67%	83%	79%	78%	58%	83%	75%	6	Yes
**US-ICSI (2018)** *	89%	56%	75%	100%	46%	100%	78%	6	Yes
**Peru (2018)**	44%	39%	48%	56%	4%	75%	44%	3	No
**US-AOPT (2021)** *	83%	83%	56%	94%	50%	100%	78%	7	Yes
**US-VA/DoD (2022)** *	72%	89%	71%	83%	63%	100%	80%	7	Yes
**US-NASS (2020)** *	72%	61%	81%	94%	46%	100%	76%	6	Yes
**US-ACOEM (2020)**	78%	44%	65%	89%	54%	92%	70%	5	Yes, with mod.
**US-ACP (2017)**	89%	56%	60%	78%	42%	100%	71%	5	Yes, with mod.
**Austria (2018)**	61%	28%	58%	94%	50%	83%	63%	5	Yes, with mod.
**Canada-TOP (2017)**	67%	28%	33%	83%	50%	33%	49%	4	Yes, with mod.
**Germany (2017)**	61%	72%	56%	94%	38%	83%	67%	5	Yes, with mod.
**Philippines (2017)**	78%	50%	77%	83%	33%	83%	67%	5	Yes, with mod.
**Qatar (2020)**	44%	67%	63%	83%	42%	83%	64%	5	Yes, with mod.
**Belgium (2017)** *	94%	78%	77%	89%	79%	100%	86%	7	Yes
**UK (2020)** *	78%	67%	63%	89%	58%	100%	76%	6	Yes
**France (2021)**	56%	56%	56%	61%	29%	100%	60%	5	Yes, with mod.
**Netherlands-NHG (2017)**	72%	56%	54%	72%	29%	58%	57%	4	No
**Netherlands-KNGF (2021)**	78%	61%	52%	61%	54%	8%	52%	4	No
**Russia-RSSP (2019)**	44%	28%	15%	56%	42%	67%	42%	3	No
**Canada-PEER (2022)**	72%	33%	40%	61%	46%	42%	49%	4	No
**Japan (2021)**	61%	44%	48%	78%	38%	83%	59%	4	Yes, with mod.
**Scotland (2019)**	83%	83%	60%	78%	58%	83%	74%	5	Yes, with mod.
**Median**	72%	56%	59%	83%	46%	83%	67%	/	/
**Interquartile range**	17%	26.75%	17%	15.5%	15%	23%	18.25%	/	/

* Defined as high-quality guidelines

### Treatment recommendations

All recommendations across CPGs are listed in Supplementary Material 3. The most critical therapeutic recommendations of CPGs for managing acute LBP, subacute LBP, chronic LBP, and unspecified duration of LBP are categorised as education, psychological therapy, exercise therapy, electrotherapy, manual therapy, and medication. Each type of back pain category is discussed in more detail below.

### Acute LBP

**Fig. 2:**
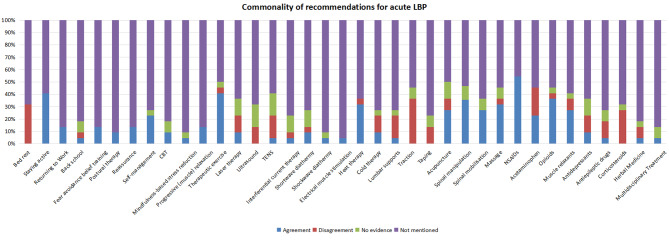
The commonality of therapeutic recommendations for patients with acute LBP

Thirteen CPGs discussed the management of acute LBP. They recommended non-steroidal anti-inflammatory drugs (NSAIDs) (*n* = 12) [19–24, 26–28, 30–32], therapeutic exercise (*n* = 9) [19, 21–23, 25, 27, 30–32], staying active (*n* = 9) [19, 22–26, 28, 31, 32], spinal manipulation (*n* = 8) [20, 22–24, 27, 30–32], opioids (*n* = 8) [19, 23, 26–28, 30–32], heat therapy (*n* = 7) [20, 22, 24, 27, 28, 30, 31], massage (*n* = 7) [20, 23, 25, 27, 30–32], acupuncture (*n* = 6) [19, 20, 22, 27, 30, 31] muscle relaxants (*n* = 6) [20, 22, 26, 27, 31, 32], spinal mobilisation (*n* = 6) [19, 25, 27, 30–32], self-management (*n* = 5) [19, 22, 25, 28, 32], paracetamol (*n* = 5) [19, 22, 28, 31, 32], returning to work (*n* = 3) [22, 28, 32], progressive muscle relaxation (*n* = 3) [27, 30, 32], reassurance (*n* = 3) [19, 22, 28], fear-avoidance belief training (*n* = 3) [23, 24, 28], Cognitive Behavioural Therapy (CBT) (*n* = 2) [19, 32], postural therapy (*n* = 2) [23, 31], laser therapy (*n* = 2) [20, 31], shortwave diathermy (*n* = 2) [27, 31], cold therapy (*n* = 2) [22, 31], antidepressants (*n* = 2) [23, 31]. One CPG recommended mindfulness-based stress reduction [32], transcutaneous electrical nerve stimulation (TENS) [27], lumbar supports [31], back school [31], interferential current therapy [31], electrical muscle stimulation [20], antiepileptic drugs [31], herbal medicine [31], shockwave diathermy [31], back school [31], and multidisciplinary treatment [25] (Fig. 2).

### Subacute LBP

**Fig. 3:**
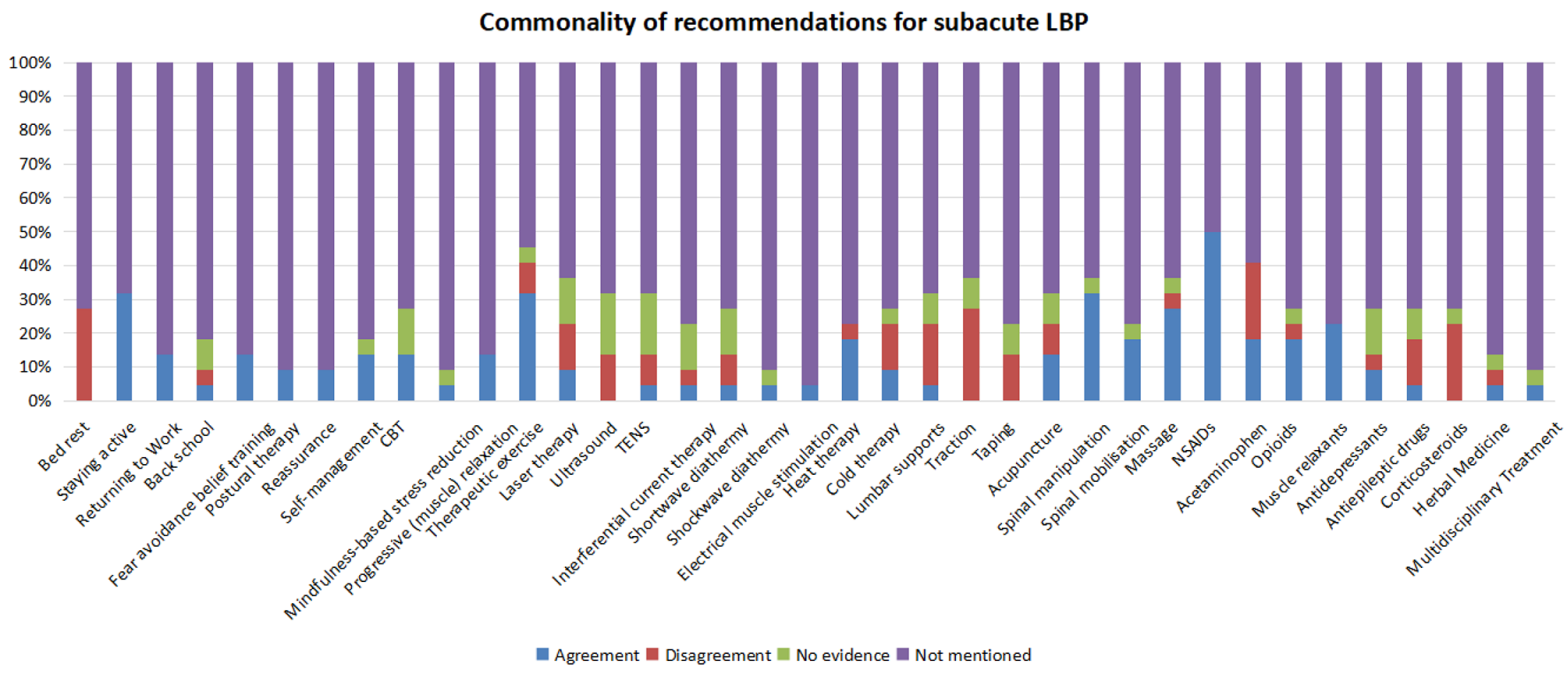
The commonality of therapeutic recommendations for patients with subacute LBP

Eleven CPGs targeted the management of subacute LBP. They recommended NSAIDs (*n* = 11) [20–24, 26–28, 30–32], staying active (*n* = 7) [22–24, 26, 28, 31, 32], therapeutic exercise (*n* = 7) [21–23, 27, 28, 30, 31], spinal manipulation (*n* = 7) [20, 22, 23, 27, 30–32], massage (*n* = 6) [20, 23, 27, 30–32], muscle relaxants (*n* = 5) [20, 22, 26, 31, 32], heat therapy (*n* = 4) [20, 22, 30, 31], spinal mobilisation (*n* = 4) [27, 30–32], paracetamol (*n* = 4) [22, 28, 31, 32], opioids (*n* = 4) [26, 28, 31, 32], returning to work (*n* = 3) [22, 28, 32], self-management (*n* = 3) [26, 28, 32], CBT (*n* = 3) [27, 30, 32], acupuncture (*n* = 3) [20, 22, 31], fear-avoidance belief training (*n* = 3) [23, 24, 28], progressive muscle relaxation (*n* = 3) [27, 30, 32], postural therapy (*n* = 2) [23, 31], reassurance (*n* = 2) [22, 27], laser therapy (*n* = 2) [20, 31], cold therapy (*n* = 2) [22, 31], and antidepressants (*n* = 2) [23, 31]. One CPG recommended back school [31], mindfulness-based stress reduction [32], TENS [31], interferential current therapy [31], shortwave diathermy [31], shockwave diathermy [31], electrical muscle stimulation [20], lumbar supports [31], antiepileptic drugs [31], herbal medicine [31], and multidisciplinary treatment [28] (Fig. 3).

### Chronic LBP

**Fig. 4:**
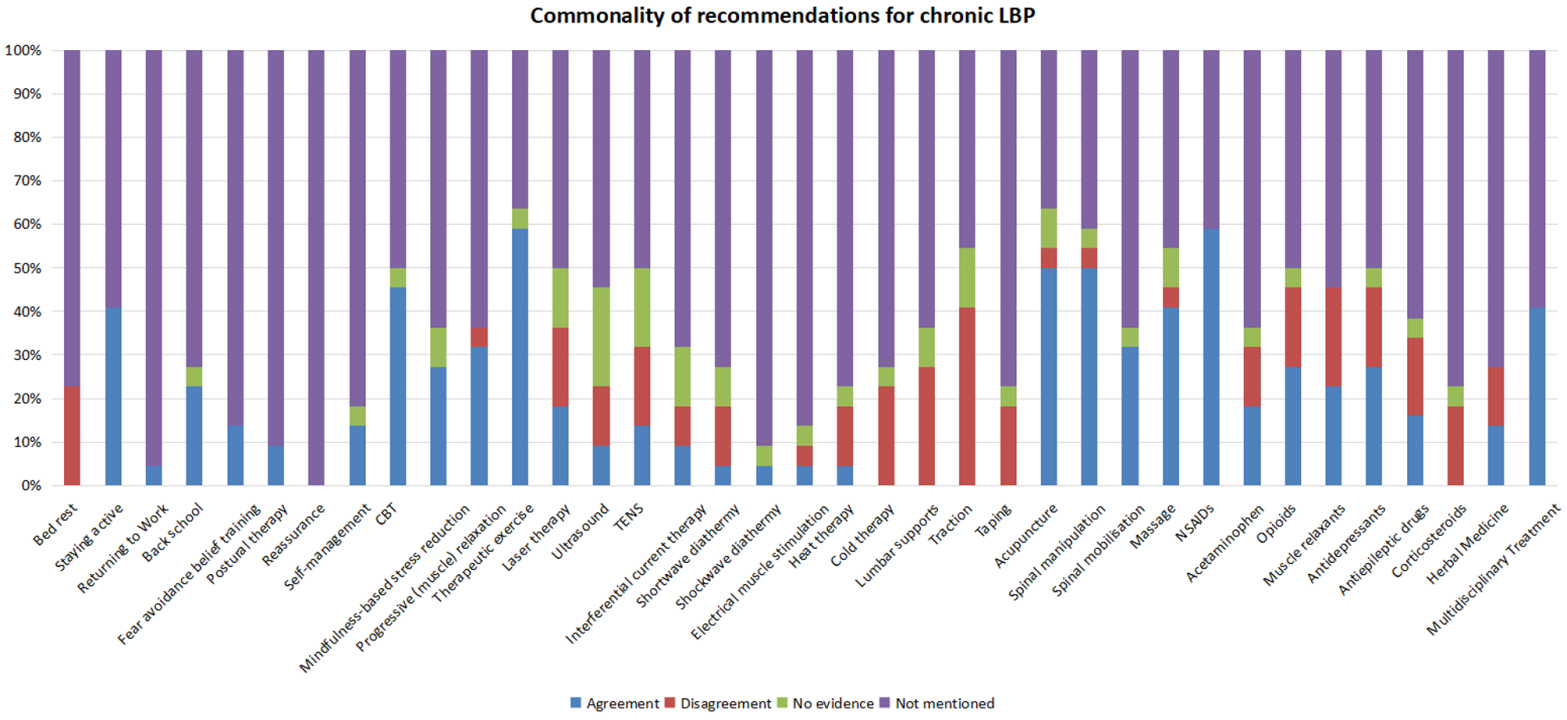
The commonality of therapeutic recommendations for patients with chronic LBP

Fourteen CPGs focused on the management of chronic LBP. They recommended therapeutic exercise (*n* = 13) [20, 21, 23, 25, 27–32, 38–40], NSAIDS (*n* = 13) [20, 21, 23, 24, 27–32, 38–40], acupuncture (*n* = 11) [20, 21, 23, 25, 27, 28, 30, 31, 38–40], spinal manipulation (*n* = 11) [21, 23, 24, 20, 27, 29–32, 38, 40], CBT (*n* = 10) [20, 21, 27–30, 32, 38–40], massage (*n* = 9) [23, 25, 27, 28, 30–32, 38, 40], staying active (*n* = 9) [23–25, 28, 29, 31, 32, 39, 40], multidisciplinary treatment (*n* = 9) [20, 21, 25, 27, 28, 32, 38–40], progressive muscle relaxation (*n* = 7) [20, 27, 28, 30, 32, 38, 40], spinal mobilisation (*n* = 7) [21, 25, 27, 30–32, 40], opioids (*n* = 6) [20, 24, 27, 30, 31, 40], antidepressants (*n* = 6) [23, 27, 28, 30, 31, 38], mindfulness-based stress reduction (*n* = 6) [20, 29, 32, 38–40], muscle relaxants (*n* = 5) [27, 28, 31, 32, 38], back school (*n* = 5) [24, 27, 30, 31, 38], laser therapy (*n* = 4) [20, 27, 31, 40], paracetamol (*n* = 4) [31, 32, 39, 40], fear-avoidance belief training (*n* = 3) [23, 24, 28], self-management (*n* = 3) [28, 32, 40], TENS (*n* = 3) [23, 27, 40], antiepileptic drugs (*n* = 3) [31, 39, 40], herbal medicine (*n* = 3) [28, 31, 39], postural therapy (*n* = 2) [23, 31], ultrasound (*n* = 2) [27, 31], and interferential current therapy (*n* = 2) [27, 31]. One CPG recommended returning to work [32], shortwave diathermy [27], shockwave diathermy [31], electrical muscle stimulation [27], and heat therapy [30] (Fig. 4).

### Unspecified duration of LBP

**Fig. 5:**
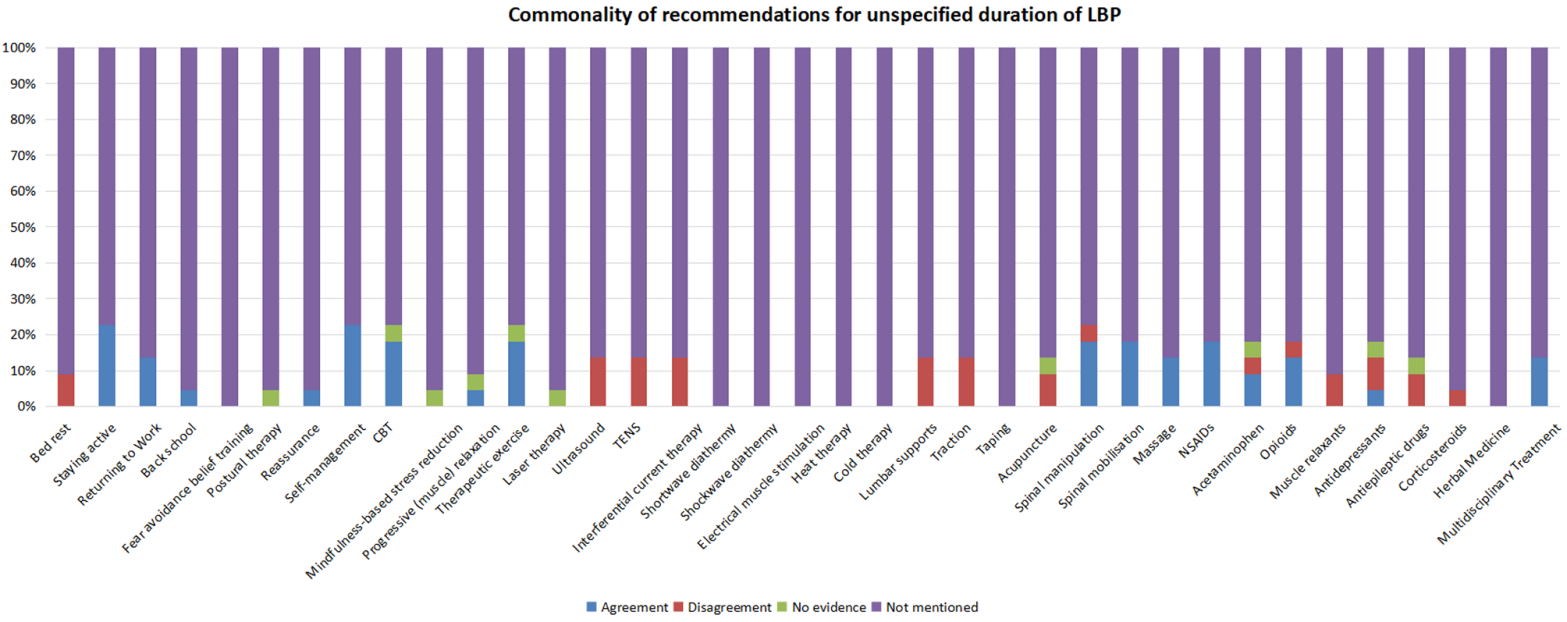
The commonality of therapeutic recommendations for patients with unspecified duration LBP

Five CPGs focused on the management of LBP of unspecified duration. They recommended staying active (*n* = 5) [33–37], self-management (*n* = 5) [33–37], NSAIDs (*n* = 4) [33–36], therapeutic exercise (*n* = 4) [33–35, 37], spinal manipulation (*n* = 4) [33–35, 37], spinal mobilisation (*n* = 4) [33–35, 37], CBT (*n* = 4) [33–35, 37], returning to work (*n* = 3) [33–35], opioids (*n* = 3) [33–35], multidisciplinary treatment (*n* = 3) [33, 35, 36], massage (*n* = 3) [33, 34, 37], and paracetamol (*n* = 2) [33, 35]. One CPG recommended back school [33], progressive muscle relaxation [37], antidepressants [35], and reassurance [33] (Fig. 5).

## Discussion

### Summary of key findings

This study included 22 relevant CPGs on the management of non-specific LBP from the last six years. Recommendations from these guidelines addressed most non-pharmacological and pharmacological treatments used in the management of acute, subacute, and chronic LBP. Key recommendations are placed on active treatments, including education, exercise, staying active, avoiding bed rest, and self-management. Guidelines also encourage treatments targeting psychosocial factors. The findings of this review are consistent with recommendations in The Lancet back pain series, which advocated the use of non-pharmacological approaches to manage low back pain [41]. The treatment options identified in this study are also similar to two recent systematic reviews of clinical practice guidelines for LBP. They summarised that current CPGs place greater emphasis on self-management, advice and education, physical and psychological treatments, and less emphasis on pharmacological and surgical options [11, 42].

### The differences in the duration of symptoms

Differences in the duration of symptoms for classifying acute, subacute, and chronic LBP were evident in our review. Chronic LBP is generally accepted in the guidelines as lasting more than 12 weeks. However, the distinction between acute and subacute LBP was variable, with definitions ranging from less than four to less than 12 weeks. This variability highlights a degree of uncertainty in the definition of acute and subacute LBP. Dunn et al.’s work illustrated that LBP is not a self-contained condition but often presents with recurrent patterns over time, challenging the traditional categorisation into acute and chronic stages [43]. Some studies also showed that pain typically improves within 4–6 weeks of an initial acute episode, but a clear demarcation between acute and subacute phases is still lacking [5, 44]. Reflecting this ambiguity, the recently updated USA-AOPT guideline revised its classification of LBP, transitioning from distinguishing between acute, subacute, and chronic stages in its previous version to only differentiating between acute and chronic stages [25].

### Quality assessment of CPGs using the AGREE II tool

In our detailed analysis of CPGs using the AGREE II tool, we observed notably lower scores in the domains of stakeholder engagement, development rigour, and applicability, which potentially impact the overall recommendations of these guidelines. Our findings are consistent with quality assessments of previous CPGs for rehabilitation [45].

The ‘Rigor of Development’ domain, in particular, is concerning when scored low, as it suggests that recommendations may lack a solid foundation in strong scientific evidence, thereby affecting the reliability and trustworthiness of recommendations [46]. A critical component of this domain is the systematic search for evidence (“Item 7: Systematic methods were used to search for evidence”), crucial for guaranteeing that the CPGs are formulated based on a thorough and methodical approach. However, the strategies used for deriving evidence were poorly reported, particularly the lack of detail about the search timeframe. The time gap from the conclusion of the literature search to the publication of the CPGs report ranged between 10 and 32 months [13]. Garcia et al. suggested that a review period exceeding three years for guidelines could result in recommendations becoming outdated by the time of publication [47]. Furthermore, few CPGs outline the conditions for their updates, such as conducting updates every two years.

### Discrepancies in the recommendation across the guidelines

In various fields of clinical expertise, the guidelines consistently endorse therapeutic exercise, NSAIDs, spinal manipulation, and staying active as key treatments. This uniformity reflects a broad agreement among most guidelines on these core management strategies for LBP. There are also numerous examples where recommendations diverge, including around the use of acupuncture, electrotherapy, heat and cold therapy, and medication.

The underlying conflicting evidence may arise from guideline development groups prioritizing evidence of clinically important efficacy (vs. sham treatments) or effectiveness (vs. usual care). Efficacy can be defined as the performance of an intervention under ideal and controlled circumstances, whereas effectiveness refers to its performance under ‘real-world’ conditions [48]. Notably, the transition from efficacy in controlled trials to effectiveness in clinical practice involves several critical steps, including adjustments for patient adherence, variability in population, clinician expertise, and resource availability. These factors can lead to an overestimation of intervention effects observed in efficacy trials when applied in everyday clinical settings [49]. In this review, some guidelines recommended acupuncture [20, 22, 31] as a therapeutic option, while others do not [32, 34]. Guidelines recommending acupuncture may have prioritised evidence compared to sham or placebo therapy rather than usual care [50].

Discrepancies in recommendations may also result from using either high-quality scientific evidence or best practice, or a combination of both. Care strategies for LBP in clinical practice are not always aligned with the best evidence and are sometimes contradictory [41, 51]. Laser therapy is considered appropriate for people with LBP in some guidelines [20, 31], but not in others [23, 24, 30]. A Cochrane review showed statistically significant but clinically unimportant pain relief for laser therapy for low back pain [52]. Guidelines recommending laser therapy in this review may only have considered high-quality scientific evidence rather than incorporating the clinical benefit. Furthermore, the reasons for differences in heat and cold therapy are probably related to insufficient evidence, leaving the committees with room for interpretation. This is substantiated by a systematic review that stated that there was insufficient data to draw firm conclusions on the effect of superficial heat and cold therapy for LBP [53].

Recommendations about the prescription of NSAIDs remain consistent, and most guidelines recommend it as the first or second option. Further recommendations about other drugs like paracetamol, opioids, muscle relaxants and antidepressants vary considerably. Part of the variation in recommendations regarding pharmacological options might reflect different medical practices across countries. The inconsistent recommendations in pharmacological interventions from USA-VA/DoD [21], Austria [27], Canada [28], Germany [30], and the UK [34] might also be attributable to the small benefit on the management of LBP as well as potential side effects, such as gastric disturbance or physical dependence [54, 55]. A review also found insufficient evidence to identify one medication as offering a clear overall net advantage because of complex tradeoffs between benefits and harms [56]. Thus, medication recommendations are likely to depend on how guideline development groups prioritise the importance of these benefits and harms.

### Overlap in the current guidelines

Although guidelines provide therapeutic recommendations according to the duration of LBP, explicit recommendations are often ambiguous, with some more general and others more detailed. There is an overlap across recommendations in the current guidelines, which often use different ways to recommend the same interventions.

**Fig. 6:**
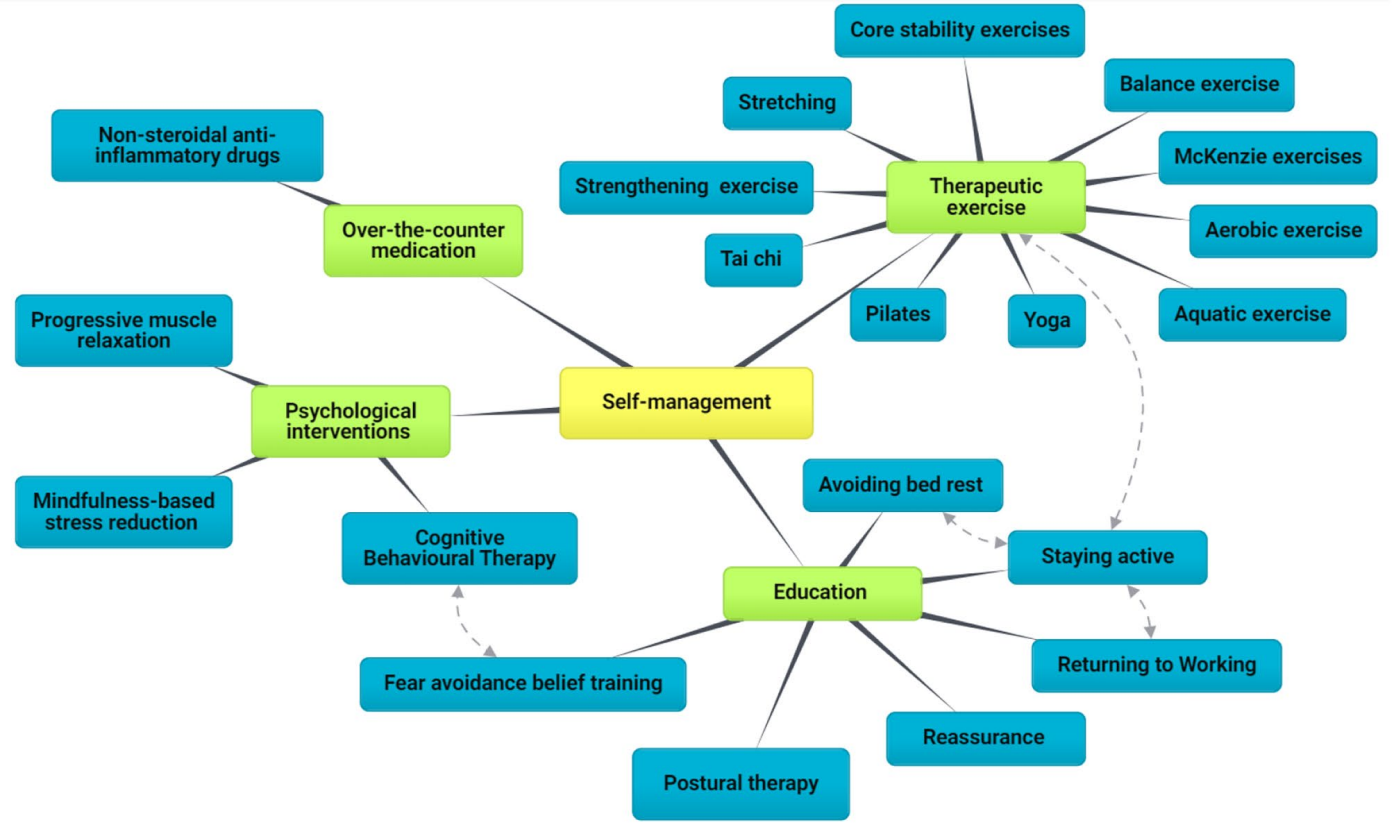
Mind map of self-management and its components. The different colour codes represent a hierarchical classification of interventions (yellow > green > blue). The dotted lines represent the possible overlap between the two interventions

The biopsychosocial model has been strongly recommended for LBP self-management [57, 58], focusing on physical, psychological, educational, and work-related components. Some interventions advocated in the guidelines could be part of a self-management approach, such as unsupervised exercise, staying active, postural therapy, CBT, and over-the-counter medications. However, they are not “branded” as self-management in the guidelines. The self-management frequently suggested by guidelines is described more as a facilitator to encourage patients to take responsibility for their symptoms, such as increasing their knowledge about the condition and their ability to continue with normal activities rather than clear goals and specific content [20, 22, 32, 35, 37, 40]. More specifically, advice to stay active, early return to work, and avoidance of bed rest are core recommendations across guidelines. However, ‘staying active’ is a broad concept and may involve avoiding bed rest and staying at work [21–23, 33, 34, 36] or it may relate to physical activity and therapeutic exercise [19, 26, 28]. With respect to the psychological treatment of LBP, CBT usually aims to help manage negative thoughts, feelings, and maladaptive health behaviours. Consequently, educational materials on fear avoidance were often confused with CBT [23, 24, 28] (Fig. 6).

In addition, spinal manipulation and spinal mobilization were often used interchangeably. Spinal manipulation is a high-velocity end-range technique with accurate movement performed to enhance joint mobility, and is more associated with chiropractic and osteopathy approaches [59]. In contrast, spinal mobilisation refers to a passive, slow-velocity technique with smooth and repeated movements used for flexibility improvement, and is more associated with physiotherapy practice [60]. However, most guidelines failed to clearly describe which one they recommend for managing LBP [19, 20, 22–24, 35, 39].

### Multidisciplinary biopsychosocial intervention

Regarding multidisciplinary team composition, most guidelines were developed by a panel of experts from various disciplines in at least three different medical fields. Whilst some CPGs [20, 21, 28, 32, 39, 40] recommended using a multidisciplinary or interdisciplinary rehabilitation program based on a biopsychosocial model, most LBP interventions were recommended as single interventions within the CPG rather than as holistic interventions [57]. Few guidelines recommended a holistic interdisciplinary approach combining physical, psychological, social, and/or occupational interventions, although some guidelines considered the incorporation of CBT into physical therapy or supervised exercise programmes. This suggests that management using a biopsychosocial model is not evident in CPGs.

### Implications

Given the outcomes of our analysis, the implications for healthcare practitioners are that there is overlap globally in recommendations, particularly for active treatments such as exercise, for the management and treatment of LBP. Although beyond the scope of this study, it is reasonable to reflect on how CPGs might consider involvement of a multidisciplinary team in their composition and incorporation of a biopsychosocial approach to provide a holistic perspective on LBP management. Additionally, adhering to a rigorous development process that emphasizes transparency and incorporates findings from the latest clinical trials can ensure that recommendations of CPGs are both relevant to the needs of the intended users and consistently reflect the most up-to-date scientific insights.

### Limitations

This study acknowledges certain limitations in comprehensively addressing the disparities in LBP management approaches that arise from resource availability, population demands and cultural and healthcare system differences. Traditional practices like acupuncture in Eastern cultures versus pharmacological treatments in Western countries reflect varying medical traditions that can lead to conflicting LBP guideline recommendations. Moreover, variations in healthcare systems, from state-provided models focusing on universal accessibility to private systems that may prioritise more costly treatments, along with the differing roles of primary care across the globe, may significantly influence the development of LBP management guidelines. Consequently, this study might not completely capture the full spectrum of these diverse management strategies.

Further, in terms of the quality assessment of included guidelines, the AGREE II training recommends that each guideline be assessed by at least two appraisers. In this review, an independent reviewer appraised all guidelines, and two other research members discussed discrepancies until a consensus was reached. This may impact the reliability of the assessments. Another limitation is the geographic representation of the included guidelines, predominantly from North America and Europe. This may be partly explained by the fact that only clinical guidelines published within the past 6 years were included in this review, potentially overlooking broader global perspectives in LBP management.

## Conclusion

This review identified 22 CPGs published between 2017 and 2022 that provide recommendations for all significant aspects of the management of LBP. Methods for conducting these CPGs varied, but most were of middle and high methodological quality. Across a global sample, current CPGs generally had similar recommendations for moving from passive bed rest and medication to active therapies. However, some heterogeneities were found among the recommendations in the CPGs of LBP due to the different approaches used in their construction.

### Electronic supplementary material

Below is the link to the electronic supplementary material.

Supplementary Material 1

Supplementary Material 2

Supplementary Material 3

## Data Availability

All data included in this study are available from the corresponding author upon reasonable request.

## Declarations

### Transparency

The authors confirm that the submitted manuscript is an honest, accurate and transparent account of the study being reported. No important aspects of the study have been omitted.
