# Supplementary material for: Recent clinical practice guidelines for the management of low back pain: a global comparison
Source: BMC Musculoskelet Disord. 2024 May 1;25:344. doi: 10.1186/s12891-024-07468-0 (PMC11061926; doi:10.1186/s12891-024-07468-0)
Supplement: Supplementary file 3 — Supplementary Material 3 [file 12891_2024_7468_MOESM3_ESM.pdf]

Search strategies

Search strategy for medical databases

Ovid MEDLINE(R) ALL <1946 to June 17, 2023>

1 low back pain.mp. or Back Pain/ or Low Back Pain/
2 lower back pain.mp. or Low Back Pain/
3 chronic pain.mp. or Chronic Pain/
4 clinical practice guideline.mp. or Practice Guideline/
5 guideline.mp. or Guideline/
6 recommendation.mp.
7 1 or 2 or 3
8 4 or 5 or 6
9 7 and 8
10 limit 9 to yr="2015 - 2022"

Embase Classic+Embase <1947 to 2023 June 17>

1 low back pain.mp. or low back pain/
2 lower back pain.mp. or low back pain/
3 back pain.mp. or backache/ 142558
4 chronic pain.mp. or chronic pain/
5 clinical practice guideline.mp. or practice guideline/
6 guideline.mp. or practice guideline/
7 recommendation.mp. or practice guideline/
8 1 or 2 or 3 or 4
9 5 or 6 or 7
10 8 and 9
11 limit 10 to yr="2015 - 2022"

CINAHL

S1 (MH "Low Back Pain") OR "lower back pain" OR (MH "Back Pain") OR (MH "Chronic Pain")
S2 (MH "Practice Guidelines") OR "clinical practice guideline"
S3 (MH "Practice Guidelines") OR "guideline"
S4 "recommendation"
S5 S2 OR S3 OR S4
S6 S1 AND S5
S7 limit S6 to (yr="2015 - 2022")

PEDro

Back pain; Practise guidelines (filtering: Since 2016)

Main guideline organisation databases

AHRQ Agency of Healthcare Research and Quality-National Guideline Clearinghouse (NGC) (https://www.ahrq.gov);
Guidelines International Network (https://www.g-i-n.net/);
Trip Medical Database (https://www.tripdatabase.com);
Agency for Clinical Innovation (https://aci.health.nsw.gov.au);
World Health Organization (https://www.who.int);
Latin American and Caribbean Health Sciences Literature (https://lilacs.bvsalud.org/);
National Institute for Clinical Excellence-NICE (https://www.nice.org.uk/);
Scottish Intercollegiate Guidelines Network-SIGN (https://www.sign.ac.uk/)
